# Comparing Two Methods of Tablet Manipulation to Adjust the Warfarin Dose in Paediatric Care

**DOI:** 10.3390/pharmaceutics12040375

**Published:** 2020-04-18

**Authors:** Jørgen Brustugun, Elisabeth Birkedal Aas, Ingunn Tho, Kathrin Bjerknes

**Affiliations:** 1 Hospital Pharmacy Enterprise, South Eastern Norway, 0050 Oslo, Norway; elisabethba@hotmail.com (E.B.A); Kathrin.bjerknes@sykehusapotekene.no (K.B.); 2 Department of Pharmacy, University of Oslo, 0316 Oslo, Norway

**Keywords:** child-adjusted dose, fraction dose, off-label, generics, patient safety

## Abstract

Tablets containing prescribed doses are not always available, and this is of particular importance in paediatric care where suitable age-appropriate formulations are generally lacking. To obtain a child-adjusted dose, tablets are manipulated in several ways; e.g., they may be dispersed in water before a fraction is extracted, or they may be split before the resulting fragment is dispersed. In this study, the accuracy attained through these manipulation methods was investigated for two generic tablets containing the anticoagulant warfarin. Tablets were dispersed in water (10 mL) before a fraction (10%) was withdrawn, alternatively tablets were split in half or quarter fragments before the fragments were dispersed in water. To investigate the contribution of variability from the different steps in the manipulation processes, the amount of warfarin recovered from the various dispersions was determined, as was the accuracy of the splitting. A validated UHPLC-method was used for quantitative determination of warfarin. Splitting of the tablets could result in deviation >30% from the ideal, theoretical weight. The amount of drug substance extracted as a fraction from the dispersed tablets deviated no more than 10% from the intended amount. To obtain the most accurate child-adjusted fraction dose of warfarin, the tablets investigated in this study should be dispersed and the desired proportion extracted. Practices that involve splitting tablets are likely to increase the variation, and should be avoided.

## 1. Introduction

Drugs in appropriate dosage form, or which contain a suitable dose for small children, are frequently lacking in peadiatric care [1,2,3]. For this reason, drugs are used off-label—i.e., outside what is approved by governing bodies [3,4]. Despite several efforts made to improve the situation, e.g., through the European Pediatric Regulation of 2007 [5], the practice is still considered an important public health issue [6]—and seems to be increasing, if anything [7]. One type of off-label handling is manipulation of drug form (e.g., splitting and crushing of tablets, opening of capsules, and dispersion of the resulting powders) [8]. It was recently found that in 17% of all oral administrations of drugs to hospitalized children (0–18 years) the drug form was manipulated prior to administration [9]. Tablets were involved in the majority of the manipulations and a fraction of the tablet was administered in approximately half the cases. In such instances, the field of pharmaceutics can aid the bedside practitioner in obtaining the desired clinical result, and avoid side effects.

Tablet manipulation may be performed for several reasons, and several manipulation methods are available: “tablet splitting, dispersing fragments” and “extraction of a proportion from a dispersed tablet”, are two examples. Splitting is the recommended method outlined in the MODRIC guideline (“Manipulation of drugs required in children”) [10] but fraction extraction is a method frequently used [10]. Considerable variation may be introduced, however, both by splitting [11,12] and fraction extraction [13,14], and the variations could possibly be tied to differences in formulation. Regarding tablet splitting, Elliott et al. [12] found that only three of eight investigated tablets met the European Phamacopoeia limit for tablet halves upon splitting, judged by weight, and the differences between the eight tablets investigated were considerable. Regarding the fraction extraction method, substantial variation in the drug amount obtained by manipulation has also been demonstrated for four different tablets containing aspirin (one chewable, one conventional, and two dispersible tablets). The mean recovery of fractions varied from 34% to 96% of that intended when tablets were dispersed and a proportion (20% of a full tablet) was withdrawn [14]. As different tablets behave so differently with regards to manipulation, questions arise. Do certain manipulation methods suit certain active ingredients or tablet formulations? Can one standard manipulation method, suitable for all tablets and substances, be established? The evidence base to answer these questions seems to be lacking.

The practice of splitting of tablets has previously been investigated in several studies [11,12,15]. However, manipulations beyond the splitting (e.g., dispersion of the fragment to aid administration), and using alternative manipulation methods (e.g., dispersing full tablets and extracting a fraction), has, to our knowledge, received little—if any—attention, especially when drug formulation and characteristics of the active ingredient (e.g., regarding solubility and pKa) are also taken into consideration. Comparing results from different tablets containing the same drug substance could aid in establishing recommendations for the off-label manipulation of tablets. Furthermore, comparing results obtained using different manipulation methods, and for different drug substances, could expand the evidence base for such recommendations.

In previous studies, focusing on aspirin tablets, we hypothesized that aspirin, being “slightly soluble” in water [16], and also being available in tablets in higher doses (e.g., 75–500 mg), could cause dose inaccuracies through sedimentation in several manipulation methods [14]. Warfarin sodium is another anticoagulant drug, differing from aspirin due to its very good water solubility [16] and lower dose requirement in tablets (e.g., 2.5 mg). Furthermore, the risks associated with warfarin therapy should be well known. Both the pharmacokinetics and pharmacodynamics of warfarin are affected by multiple interpersonal variations [17], and because of this, individualized doses need to be frequently prepared—e.g., through tablet splitting [18]. In paediatric care, warfarin has been given in doses of 0.1–0.3 mg kg^−1^ [19] to obtain a suitable effect—as monitored by international normalized ratios (INRs), and INR ranges of 1.4–1.8, 2.0–3.0, and 2.5–3.5 have been targeted. Since children are a highly heterogeneous group with regard to weight, and the content of tablets is fixed (e.g., containing 2.5 mg), manipulation of tablets in this population is common. However, few ideal manipulation methods are available, as the manipulation of tablets has been stated to lead to inconsistent dosing [20], and the reliability of liquid warfarin formulations has been questioned [21]. Considering the risks involved in warfarin use and the importance of obtaining accurate doses, knowledge about the suitability of different manipulation methods for different tablets is of interest. With characteristics very different to aspirin (e.g., regarding solubility and therapeutic window), the manipulation of warfarin tablets could also add new information of a more general nature on the topic of tablet manipulation.

In the study at hand, it was of interest to investigate whether the manipulation method and choice of tablet would affect the accuracy of the child-adjusted doses obtained for warfarin sodium. The two brands of generic tablets containing warfarin sodium licensed in Norway were compared with regards to precision and accuracy for tablet fractions obtained upon manipulation. Concerning manipulation methods, entire tablets were either dispersed in liquid before a fraction was withdrawn or tablets were split and a fragment (half or quarter) was dispersed directly in an oral syringe or a medicine measure. The methods investigated were observed in use in manipulation on the hospital wards.

## 2. Materials and Methods

### 2.1. Materials

Warfarin sodium (92.1%) and indigo carmine were provided by Sigma-Aldrich Co., St. Louis, MO, USA. Potassium dihydrogen phosphate was provided by Merck KGaA, Darmstadt, Germany. Methanol (HPLC-grade) was provided by Rathburn Chemicals Ltd., Walkerburn, Scotland, and EMD Millipore Corporation, Billerica, USA. Hydrogen peroxide (30%) was from VWR AnalaR Normapur, VWR International S.A.S., Fontenaysous-Boise, France. For adjusting pH, sodium hydroxide (2M) and hydrochloric acid (2M), both from Oslo Hospital Pharmacy, Oslo, Norway were used.

The tablets investigated were Marevan^®^ 2.5 mg, Takeda AS, Asker, Norway, (batch: 385922, 386084) and Warfarin Orion^®^ 2.5 mg, Orion Pharma, Espoo, Finland, (batch: 1740470). The SmPC (Summary of Product Characteristics) states that Warfarin Orion^®^ can be split into two equal parts; statement regarding suitability for splitting is not given for Marevan^®^. Both tablets are scored with a cross. Further characteristics for the two tablets are given in Table 1.

### 2.2. Analytical Method

For the determination of the recovered amount of drug substance upon manipulation, a UHPLC-method was used. *Sample preparation:* Prior to determination, samples were transferred to a 50 mL volumetric flask containing 40 mL phosphate buffer, pH 7.4. Upon a resting period of about 5 min the flask was inverted 25 times before phosphate buffer was added to the 50 mL mark. The volumetric flask was again inverted three times before approximately 10 mL of the sample solution was centrifuged (2500 rpm, 5 min). The supernatant was transferred undiluted to an injector vial, or when necessary diluted in phosphate buffer pH 7.4 to the theoretical target concentration (i.e., 100% recovered) for quantification (5 µg mL^−1^).

A standard curve was prepared from a solution of 10 mg of warfarin sodium in 200 mL phosphate buffer pH 7.4. This solution was diluted to concentrations from 2.3 to 34.5 µg mL^−1^.

The quantification was performed on an UHPLC-system with DAD detection (304 nm) from Shimadzu Corporation, Kyoto, Japan. Separation was performed on a Inertsil C8-3, 2 µm, 2.1 × 100 mm column from GL Sciences Inc., Tokyo, Japan, with a mobile phase consisting of methanol:water: glacial acetic acid (68:32:1, *v/v*). Sample volume was 2 μL, flow rate was 0.2 mL min^−1^, sample cooler temperature was 4 °C, and the column oven was set to 40 °C. The linearity *r*^2^ was >0.999 for the range 2.3–34.5 µg mL^−1^; the system precision was 0.1%, the intra-day precision was <0.4% (rsd for each of *n* = 3 days) and the inter-day precision was 1.2% (rsd over 3 days). The recovery was established by determining warfarin in tablet powder equal to 50% of a tablet, 75%, 100%, 100%+25% warfarin sodium substance, and 100%+50% warfarin sodium substance. The linearity for the five samples was *r*^2^ = 0.997.

The specificity for warfarin was demonstrated with regards to the colorant indigocarmine, and for degradation products obtained in stress studies employing heat, hydrogen peroxide (3%) or alkaline conditions. All peaks detected were separated from warfarin with Rs ≥ 2.8. Sample stability was established for at least 24 h.

### 2.3. Instruments

The experiments utilized a Sartorius CPA225D-0CE analytical balance (Sartorius AG, Göttingen, Germany), a Metrohm 691 pH Meter (Metrohm AG, Herisau, Switzerland) and a Branson 5510 ultrasonic bath (Branson Ultrasonics B.V., Eemnes, The Netherlands). For the physical characterization of tablets, a TBH 125 tablet hardness tester, a Erweka TA friability tester, and a Erweka ZT3-2 disintegration tester from Erweka GmbH, Heusenstamm, Germany was employed.

### 2.4. Tablet Manipulation Devices

The oral syringes were Baxter Exactamed (1 and 5 mL) from Baxter Healthcare SA, Zürich, Switzerland. The medicine measure was a polypropylene medicine measure (D: 38 mm, H: 42 mm, 30 mL) from Hammarplast Medical AB, Lindköping, Sweden. Tablets were split using an Apro tablet splitter from Karo Pharma AS, Oslo, Norway.

### 2.5. Physical Characterization

A measure of tensile strength was obtained by measuring the breaking strength (N) of 10 tablets of each type. The diameters of the tablets were measured with a Cocraft digital caliper (0–150 mm, accuracy: 0.03 mm). The average tensile strength (N/mm^2^) with high and low values was calculated. 

The disintegration time in distilled water (35–39 °C) was recorded for six tablets using a ZT3-2 disintegration tester. The average disintegration time (s) with high and low values was calculated.

Friability was obtained for 54 tablets (constituting a total weight nearest to 6.5 g, as described in the European Pharmacopoeia 2.9.7). The tablets were placed in the drum of the Erweka TA friability tester before the drum was rotated 100 times. The tablets were weighed before and after the 100 rotations and the weight lost (%) was calculated.

### 2.6. Manipulation Studies

#### 2.6.1. Tablet Splitting

Tablets (*n* = 10) were split in halves and quarters and the weight of the fragments were recorded. The fragment weight, as per cent of total tablet weight, was calculated and the deviation from the ideal split was taken as a measure of splitting accuracy. The weight of crumbles, tablet mass not included in any half or quarter part, was calculated as weight of all half or quarter fragments subtracted from weight of whole tablet. The tablets were split using a dedicated splitting device (Apro tablet splitter).

*Splitting accuracy* was judged by the closeness of the average value obtained (%) to the theoretical halves and quarters of the tablet weight.

#### 2.6.2. Tablet Split, Fragment Dispersed in Medicine Measure

**Figure 1:**
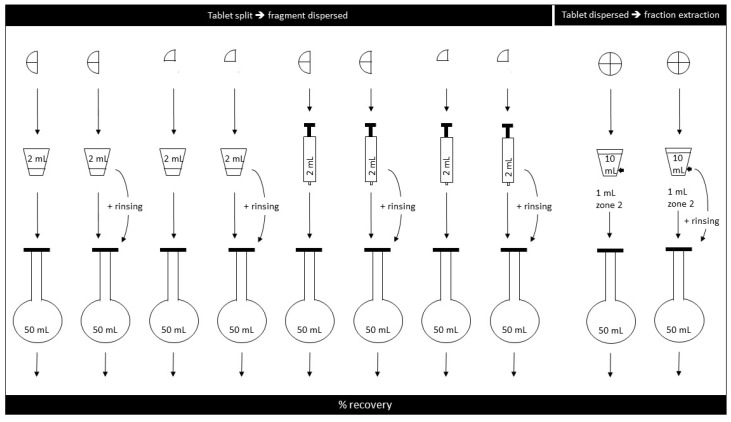
Illustration of tablet manipulations performed to obtain child-adjusted doses (n = 6).

*Half tablets*: Tablets were split in two by use of a commercially available tablet-splitting device. The largest half by weight was placed in the 30 mL medicine measure with 2 mL purified water for 8 minutes; the sample was stirred 10 s every minute. The suspension was pumped in to and out of a 5 mL syringe four times before the whole fluid volume was finally withdrawn, ejected into a 50 mL volumetric flask (Figure 1), and prepared further as described above (see *Analytical method*).

*Quarter tablet*: The experiment was repeated, but this time the tablet was split into four parts and the largest quarter fragment was dispersed.

*Effect of rinsing*: The two experiments (above) were repeated, but this time the medicine measure was rinsed with 1 mL purified water which was added to the 2 mL sample already in the volumetric flask. 

#### 2.6.3. Tablet Split, Fragment Dispersed in Oral Syringe

The experiments were performed as described above, but this time the largest half or quarter fragment was placed directly in a 5 mL oral syringe, 2 mL purified water was then drawn up (Figure 1), and the syringe was left horizontally for 8 min to allow tablet disintegration; the syringes were shaken gently 10 s very minute. The experiment was repeated with a rinsing step where 1 mL of purified water was used to rinse the syringe after first ejection. The 2 mL sample, and the 1 mL rinsing volume, when rinsing was performed, was ejected into a 50 mL volumetric flask, and samples were prepared as described above.

In experiments where tablets were split, the amount of warfarin found after manipulation was calculated relative to the actual weight of each half or quarter fragment manipulated.

#### 2.6.4. Tablet Dispersed in Water and a Fraction Withdrawn

In a 30 mL graduated plastic medicine measure, a single warfarin tablet was placed in 10 mL purified water for 8 min. The sample was subjected to stirring for 10 s every minute and prior to withdrawal of the sample the suspension was pumped into and out of the syringe four times. Samples of 1 mL were withdrawn with 1 mL syringes from the 2 mL mark of the medicine measure (“Zone 2” as outlined by Broadhurst et al. [13]). Six medicine measures, each containing one tablet, were prepared for each of the two tablet formulations. One sample was withdrawn from each medicine measure, and the warfarin content of the sample was determined in triplicate.

For each tablet type and manipulation method, six samples were prepared; three control samples consisting of tablet powder equal to one average tablet mass were analyzed for every manipulation experiment performed. The tablet powder in the control samples always came from the same batch as the tablets manipulated in the same experiment. The amount of drug substance recovered was normalized using the average value of the control samples in the experiment.

#### 2.6.5. Explanation and Definition of Terms

In this study, the *intended* dose refers to the fraction dose aimed at, i.e., the ideal half, quarter, or tenth of a tablet. The *expected* amount refers to the amount of drug substance recovered with regard to the weight of the individual tablet fragment manipulated after a tablet splitting.

Regarding *precision*, the European Pharmacopoeia [22] states that for split tablets, no fragment should deviate more than 25% from the average (*n* = 30), and only one fragment should deviate with more than 15%. Formal limits with regards to tablet fractions obtained by manipulated are not known to us, but 20% deviation has been practiced previously [14,23]. In this study, these limits are used to provide context to the acceptability of the variability introduced by different manipulations; however, considerations regarding individual drug substances should be made in addition.

*Accuracy* was defined as the closeness of the average value obtained (%) to the intended dose or expected amount. *Precession* was defined as the variation around the average value obtained, and the result is given as both lowest–highest value and standard deviation (sd).

## 3. Results

### 3.1. Tablet Characterization

The two generic tablets, Marevan^®^ and Warfarin Orion^®^, were both conventional tablets containing 2.5 mg warfarin sodium and similar excipients. Some variation in physical characteristics was observed (Table 1). The pH-value of a dispersed tablet was lower for Warfarin Orion^®^, with pH 6.5 as compared to 7.2 for Marevan^®^. These tablets also had a lower friability, higher tensile strength, and increased disintegration time as compared to Marevan^®^.

### 3.2. Control Samples

For every assay performed, the content of ground up tablet mass equal to one whole tablet (*n* = 3) was determined, with no manipulation performed. Recoveries obtained from these samples were: 96.3% (±2.8) (85.8–100.7) (Mean (sd) (lowest–highest value)) (*n* = 36) for Warfarin Orion^®^, and 97.7% (±3.6) (87.7–102.5) (*n* = 38) for Marevan^®^.

### 3.3. Splitting Accuracy

The splitting accuracy of the two tablets investigated in this study—using a dedicated tablet-splitting device—is shown in Table 2 for half and quarter fragments. The difference between fragments was smallest for Warfarin Orion^®^. The tablet mass lost to crumbling during splitting constituted at most 2.1% of the full tablet, this was found after quartering of Marevan^®^ (Table 2).

### 3.4. Dispersing Tablets in Water and Extracting A Fraction

The amount recovered upon suspending full tablets in water (10 mL) and withdrawing a fraction (1 mL), is shown in Table 3. The 1/10 tablet fraction aimed at was recovered with up to 9.2% deviation at most, and the average value for the two tablet types did not significantly differ (*p* = 0.09).

### 3.5. Dispersing Tablet Fragments in A Medicine Measure

The amount recovered upon dispersing half and quarter fragments in a medicine measure is shown in Table 4. Subjecting the same tablet fragments to the same manipulation, the results for the two tablets were not different (*p* > 0.05), but for the half tablets with no rinse performed, the recovery from Marevan^®^ was higher than from Warfarin Orion^®^ (95.8% and 90.8%, respectively, *p* = 0.009)

### 3.6. Dispersing Tablet Fragments in an Oral Syringe

The amount recovered upon dispersing half and quarter fragments in an oral syringe is shown in Table 5. The results for the two tablets were again not different (*p* > 0.05 for the same fragments subjected to the same manipulation). The exception being half tablets dispersed in the oral syringe followed by the rinse. Here the Marevan^®^ gave a higher recovery, closer to the expected value (*p* = 0.007).

## 4. Discussion

### 4.1. Manipulation of Warfain Tablets to Obtain a Fraction: Comparing Methods

The manipulation of tablets can be performed for several reasons, but often, the aim is to achieve an adjusted dose. Variability may be introduced at several points in a manipulation process, however, and in the case of this study, two standardized methods of tablet manipulation were compared with regard to the ability to produce accurate and precise amounts of warfarin: “split-and-disperse”, and “fraction-extraction”. In a fraction-extraction manipulation, variability is contributed by the disintegration and dissolution of the tablet and active ingredient, respectively, the extraction of tablet dispersion into the oral syringe, and the delivery from the syringe. The variability contributed by the sum of these steps was investigated as one process and is presented in Table 3. The split-then-disperse method, on the other hand, was investigated as a two-step process, consisting of the splitting (Table 2), followed by the dispersion and delivery of the fragment, either using a medicine measure (Table 4) or dispersing the fragment directly in the oral syringe (Table 4 and Table 5). Table 3 shows that, using fraction-extraction, the average amounts of warfarin close to 100% of that intended could be obtained. Likewise, Table 4 and Table 5 show that the recovery from tablet fragments was also fair, and that the disintegration and delivery step only contributed a modest amount of variability. Using an oral syringe for dispersion and delivery, recovered amounts on average deviated less than 6% from the expected value for the individual half or quarter fragment.

However, the splitting step in the “split-and-disperse” approach typically introduced more variability (Table 2). Quarter fragment weights could, on average, constitute 23.3–26.4% of the whole tablet weight for the tablet with the least variation, Warfarin Orion^®^, with individual fragments spanning the range 22.2–27.6%. For Marevan^®^, the variation was more pronounced, with individual quarter fragments ranging from 17.2% to 33.6% of the full tablet weight. The 33.6% fragment would theoretically contain 0.84 mg warfarin when the aim was 0.625 mg, i.e., 134% of the amount intended; the 17.2% fragment by weight would theoretically contain only 69% of the amount intended. To summarize, for Marevan^®^, the deviation from the intended value contributed by splitting alone could be >30% (i.e., 69–134%).

Deviation from the intended amount of warfarin introduced by fraction-extraction was <10% (Table 3). This value was comparable to the variability in recovery found for the dispersed split fragments (Table 4 and Table 5), and for these fragments the variability introduced by the preceding split would come in addition. Taken together, these results indicate that the most reliable manipulation method for the two warfarin tablets is the fraction-extraction—dispersing a full tablet and extracting a fraction.

### 4.2. Manipulation of Warfain Tablets to Obtain A Fraction: Considerations Regarding Physico-Chemical Properties

Warfarin sodium is very soluble in water [16] and has a pKa of 5.05 [24]. However, in its protonated free acid form, the substance is practically insoluble, and a solubility in strong acid of 4.4 mg/L has been reported [24]. In our experiments, the pH of the dispersions was 6.5 for Warfarin Orion^®^ and 7.2 for Marevan^®^ (Table 1), both heavily favoring the soluble form, but importantly, also containing a smaller fraction of the insoluble form. This insoluble fraction should theoretically be higher in dispersions of Warfarin Orion^®^ than Marevan^®^ since the pH was closer to the pKa for Warfarin Orion^®^. Based on calculations according to the Henderson–Hasselbalch equation, the theoretical content of the poorly soluble form should not exceed a few per cent, about 4% and 1%, respectively. In the manipulations performed in this study, the concentrations of warfarin sodium spanned from 250 mg L^−1^ (for a full tablet dispersed in 10 mL water) to 625 mg L^−1^ (for half a tablet dispersed in 2 mL water). For Marevan^®^, this means that even the “insoluble” protonated fraction could mostly or fully dissolve in the available liquid, at least when dispersing full tablets in 10 mL water. For Warfarin Orion^®^, with its less favorable pH when dispersed, a small fraction of protonated, undissolved warfarin must be expected in all manipulations, as the available liquid is not sufficient to fully dissolve the estimated protonated fraction. Looking closely at the results obtained, such effects of pH and solubility may possibly be observed (Table 3, Table 4 and Table 5). In most of the experiments, the accuracy in recovery for dispersed tablets and fragments was well within 20% of expected values, reflecting, perhaps, that most or all of the warfarin was dissolved, regardless of tablet type or manipulation performed. However, in the experiments where differences are observed between the two tablets, Marevan^®^ tends to give the higher value (i.e., closer to the expected value) by some few percent points, congruent with the favorable solubility at the higher pH in dispersions of Marevan^®^ (Table 3, Table 4 and Table 5).

Regarding excipients, the composition of the two tablets was similar (Table 1), and as the amounts of the individual excipients are not known, effects of single excipients on the manipulation outcomes cannot be extracted. The physical characterization, however, showed Warfarin Orion^®^ to be a less friable tablet, with higher tensile strength and longer disintegration time, compared to Marevan^®^ (Table 1). These characteristics could possibly explain the better splitting accuracy of Warfarin Orion^®^ (Table 2), and following that, a better suitability for a split-then-disperse manipulation. On the other hand, the characteristics of Warfarin Orion^®^ (e.g., through its longer disintegration time) could also possibly explain the lower amounts recovered when a medicine measure was utilized (Table 3 and Table 4)—a manipulation step where sedimentation could affect the outcome.

It is possible that the high recovery of warfarin observed from both whole tablets and tablet fragments (Table 3, Table 4 and Table 5)—perhaps caused by the good solubility of warfarin—could mask effects of excipients or physical characteristic of the tablets on manipulation outcomes. Because of this, comparing the results from the study at hand to those previously obtained from manipulation of tablets containing aspirin, a substance that is only slightly soluble, could be of value.

### 4.3. Comparing Manipulation of Warfarin Tablets to Manipulation of Aspirin Tablets

The finding that most of the warfarin tablet manipulations—except the splitting step—gave an acceptable recovery is in contrast to what was observed for tablets containing slightly soluble aspirin [14,25]. Comparing four types of aspirin tablets (one chewable, one conventional, and two dispersible), both accuracy and precision in the aspirin amount retrieved could be poor depending both on the tablet type chosen and the manipulation method used [14], e.g., for one 500 mg aspirin tablet only 3% of the dose intended was recovered when a full tablet was dispersed in 10 mL water and 1 mL was directly extracted as the simulated dose, and no more than 43% could be recovered using various mixing procedures. Splitting the tablet first, dispersing the half or quarter fragments in water in general improved accuracy, particularly if the fragment was dispersed directly in the oral syringe. A considerable variability could still be observed, however, especially when fragments were dispersed in a medicine measure [25].

For aspirin, as for warfarin, it was hypothesized that variations in solubility, resulting from differences in pH, could explain several observations. Aspirin is only slightly soluble in water (1:300), and has a pKa of 3.5 [16]. In addition, the drug content in each aspirin tablet varied, from a low value of 75 mg in Dispersible Aspirin^®^, and up to 500 mg. Particularly poor recovery after manipulation was observed for high dose aspirin tablets where the pH of the dispersion < pKa (i.e., where solubility is not favored) and where the tablets were manipulated such that sedimentation could take place, as in a medicine measure. Conversely, for the lower dosed aspirin tablets where dispersion pH > pKa (favoring solubility), dose accuracies were consistently more reliable.

As indicated, manipulating aspirin tablets could result in dispersions with pH values both above and below the pKa value of the drug substance, greatly affecting the solubility. However, the current warfarin results suggest that even more modest differences in pH relative to the pKa value of the drug substances can also be influential, as suggested by the results of this study. For dispersions of Warfarin Orion^®^, where pH is 6.5, and almost 1.5 units above the pKa (aiding solubility), an effect of incomplete dissolution can possibly be observed, e.g., for the half tablets dispersed in a medicine measure with no rinse being performed (Table 4). In this manipulation experiment, an average recovery from the fragments of 90.8% (with a lowest value of 84.7%) was observed. For the Marevan^®^ tablets, where the pH difference to pKa was more than two units, the average recovery from the fragment was higher (95.5%).

The large variability previously observed upon manipulation of tablets containing slightly soluble aspirin was not observed for the tablets investigated in this study, containing very soluble warfarin sodium. Taken together, the results points to solubility being an important factor in deciding manipulation outcomes for tablets. Because excipients can influence the pH in a tablet dispersion, and by that the solubility of the drug substance, knowledge about formulation effects can aid the clinician in choosing the most suitable manipulation method to increase patient safety when manipulation is necessary.

### 4.4. Manipulation of Warfain Tablets to Obtin a Fraction: Some Clinical Considerations Regarding Paediatric Dosing

The theoretical warfarin doses achieved by the manipulations investigated in this study would be relevant for children from below 1 kg up to 12.5 kg. To illustrate, if a tablet containing 2.5 mg warfarin was split into halves, each half theoretically containing 1.25 mg warfarin, this would provide a relevant dose for a child between 4.2 kg and 12.5 kg, given a suggested dose region of 0.1–0.3 mg kg^−1^ [19]. The extraction of a tenth of a dispersed 2.5 mg tablet (i.e., 0.25 mg) would theoretically provide a relevant dose for a child between 0.8 kg and 2.5 kg, e.g., a lower weight neonate [26]. The corresponding ages covered would range from the neonates mentioned up to toddlers, depending on health state, physical development, sex, and monitored INR.

The results in this study show that the most accurate child-adjusted doses of the investigated generic warfarin tablets can be obtained by dispersing a tablet and withdrawing a fraction (Table 3). The recovery of warfarin from split fragments also show fair accuracy (Table 4 and Table 5). However, the variability arising in the splitting process (Table 2) could be considerable: As discussed above, quarter fragments of Marevan^®^ could already vary between 69% and 134% of the ideal split, prior to any further manipulation.

The acceptability of these variations in a clinical setting is hard to determine in a definitive way, due to differences in INR-ranges aimed at, and differences between patients. However, it has been stated in literature (though without a specific paediatric focus) that there is a “reasonable linear relationship between dose and INR response during maintenance dosing”, where a “10% dose increase will result in an increase of approximately 10% in the INR” [27]. And furthermore, to maintain INR between two and three, dose changes of 10–20%, were suggested. This indicates that variations arising in the splitting process (sometimes exceeding 30%) may be clinically meaningful for the warfarin tables investigated here. The variation arising when whole tablets are dispersed and a fraction is withdrawn (not exceeding 10%), should be less clinically meaningful. 

In our experience, Marevan^®^ (2.5 mg) is the warfarin tablet most frequently used in the paediatric wards in Norway, and for these tablets, manipulation through dispersion of tablets and extraction of a proportional fraction is the safer choice. This method avoids splitting, and offers a choice of relevant doses in paediatric therapy.

### 4.5. General Considerations Ragarding Paediatric Doseing from Manipulated Tablets

The results of this study showed that, for the very soluble substance warfarin, dispersion only added a modest amount of variability. Splitting, however, could add more, resulting in variability that could be clinically meaningful. For the slightly soluble substance aspirin, investigated previously, the opposite was found to be the case, as the variability introduced in the dispersion step could exceed that of splitting. Marked variations between the different kinds of aspirin tablets were also observed.

These findings demonstrates that when tablets are manipulated to obtain a fraction, the choice of manipulation method and, indeed, tablet-type or brand, should be guided both by knowledge about the solubility of the drug substance, the pH created by both drug substance and excipients upon dispersion, and by knowledge about the variation that is introduced by tablet splitting. The preferable manipulation method is likely to be different for different tablets and substances. With this in mind, and understanding that an appreciable percentage of paediatric administrations involve the manipulation of tablets [9], the knowledge provided by the field of pharmaceutics can be of benefit when paediatric doses are administered in the clinic.

### 4.6. Limitations

In this study, one operator performed all manipulations. The operator was trained in the task and worked with dedicated attention to accuracy and precision. Thus, the results may be somewhat idealized and do not cover variation arising from different persons performing manipulations in a clinical setting.

To limit study variables, only the largest fragment was chosen for manipulation in the manipulations that involved splitting and tablet fragments. Variation arising from choosing the smaller tablet fragments is not covered in the data.

The warfarin tablets included in the study were the two available on the Norwegian market at the time of the investigation. They showed little variation regarding types of excipients used, and so variations in manipulation caused by the excipient characteristics are not well illustrated. However, the amount of the excipients, the quality of excipients (e.g., particle size), and the specifications for the various steps in the production process (e.g., granulation method or regarding force applied in the tablet press), though unknown to us, could be different for the two tablets. Indeed, the differences observed in the physical characteristics (Table 1) could suggest this to be so.

The European pharmacopoeia allows for some variation in dose between tablets, and the control samples illustrate the accuracy in warfarin content for the two tablets investigated. The true content of each manipulated tablet and tablet fragment is not known, however.

The tablets were all split using a dedicated tablet-splitting device. Using alternative splitting methods (e.g., breaking by hand or using a knife) could affect outcomes of the splitting. Variability caused by using different splitting methods is not illustrated by the current results.

## 5. Conclusions

Two generic warfarin tablets were investigated with manipulation methods suitable for use on the hospital ward in order to obtain child-adjusted doses for paediatriac care. The manipulation methods were evaluated with regard to their ability to produce accurate and precise amounts of drug substance. Less than ±10% deviation from the intended value was observed as a result of dispersing tablets and extracting a fraction. When splitting was a part of the manipulation procedure, it could add more than ±30% variation. For the two warfarin tablets investigated, dispersing full tablets and extracting a fraction, omitting splitting, is the safer choice when the manipulation of tablets cannot be avoided.

## Figures and Tables

**Table 1:** Characteristics of the two generic warfarin tablets studied, including results of physical tests.

Characteristics	Marevan^®^ «Takeda»	Warfarin Orion^®^ «Orion»
API (Content)	Warfarin sodium (2.5 mg)	Warfarin sodium (2.5 mg)
Excipients	Lactose monohydrate, Corn starch, Polyvinyl-polypyrrolidone, Calcium hydrogenphosphate dihydrate, Magnesium stearate, Indigotine (E132)	Lactose anhydrous, Corn starch, Polyvinyl-polypyrrolidone, Calcium phosphate, Magnesium stearate
Suitability for splitting ^1^	Not specified	Yes, can be split in two
Tablet weight (g) ^2^	0.121 ± 0.002	0.119 ± 0.001
Tablet dimensions (mm), d × h ^3^	7.00 × 2.73	7.04 × 2.74
pH dispersed tablet ^4^	7.2	6.5
Friability (%) ^5^	0.6	0.2
Disintegration (s) ^6^	123 (115–135)	161 (90–240)
Tensile strength (N/mm^2^) ^7^	1.43 (1.20–1.83)	1.99 (1.87–2.37)

^1^ SmPC; ^2^ Mean (g) ± sd (*n* = 10); ^3^ Diameter × height (Mean, *n* = 3; sd% < 0.7%); ^4^ pH of the suspension of one tablet suspended in 10 mL purified water; ^5^ Percent lost upon friability testing (Ph.Eur.9.2) (*n* = 54); ^6^ Average time (seconds) to disintegrate (*n* = 6, low-high); ^7^ Calculated from breaking strength (*N*), diameter (mm) and height (mm). Average values are given (*n* = 10, low-high).

**Table 2:** Splitting accuracy of the two warfarin tablets investigated. Per cent of full tablet mass is given for half and quarter fragments, from largest fragment (Fragment 1) to smallest fragment. (Mean ± sd (low to high); *n* = 10) ^1^

Tablet Fragment	Marevan^®^	Warfarin Orion^®^
Half fragment 1	52.8 ± 2.5 (49.9–57.9)	50.9 ± 0.5 (50.3–52.0)
Half fragment 2	45.7 ± 3.5 (49.6–40.1)	49.0 ± 0.5 (48.0–49.5)
Quarter fragment 1	28.1 ± 2.1 (26.3–33.6)	26.4 ± 0.7 (25.6–27.6)
Quarter fragment 2	24.4 ± 2.2 (21.5–28.6)	25.7 ± 0.7 (24.8–27.0)
Quarter fragment 3	24.0 ± 1.8 (20.4–25.8)	24.4 ± 0.8 (23.1–25.3)
Quarter fragment 4	21.3 ± 2.4 (17.2–24.5)	23.3 ± 0.8 (22.2–24.6)

^1^ Mass not included in any fragments (i.e., crumbles, etc.) represented 1.4% and 2.1% of the tablet mass for half and quarter Marevan^®^ tablets, respectively, and 0.1% and 0.2% of the tablet mass for half and quarter Warfarin Orion^®^ tablets, respectively.

**Table 3:** Extracting a fraction: Suspending a warfarin tablet in 10 mL water and extracting a 1 mL fraction ^1^. Per cent of intended value ± sd (low–high) (*n* = 6), is given.

Tablet	Per Cent of Intended Warfarin Amount (0.25 mg)
Marevan^®^ 2.5 mg	103.3 ± 4.2 (98.1–109.2)
Warfarin Orion^®^ 2.5 mg	98.2 ± 4.9 (91.4–103.3)

^1^ All samples were extracted from Zone 2 of the medicine measure. Samples were adjusted for the value of the control sample, i.e., a full tablet not manipulated, determined the same day.

**Table 4:** Dispersing a fragment: Per cent recovered for fragments of warfarin tablets dispersed in a medicine measure ^1^. Per cent of expected value ± sd (low–high value) (*n* = 6), is given ^2^.

Tablet	Half Tablet, No Rinse	Half Tablet, with Rinse	Quarter Tablet, No Rinse	Quarter Tablet with Rinse
Marevan^®^ 2.5 mg	95.8 ± 2.2 (93.3–98.9)	98.9 ± 2.8 (93.7–101.8)	98.2 ± 4.9 (89.3–103.1)	105.6 ± 3.2 (99.9–109.5)
Warfarin Orion^®^ 2.5 mg	90.8 ± 3.1 (84.7–93.1)	100.0 ± 1.1 (98.5–101.4)	99.3 ± 2.2 (95.3–101.7)	107.3 ± 3.4 (103.5–113.5)

^1^ Samples were adjusted for the value of the control sample, i.e., a full tablet not manipulated, determined the same day. ^2^ Expected with regard to the individual fragment weight. The 30 mL medicine measure contained 2 mL purified water.

**Table 5:** Dispersing a fragment: Per cent recovered for fragments of warfarin tablets dispersed in an oral syringe ^1^. Per cent of expected value ± sd (low–high value) (*n* = 6), is given ^2^.

Tablet	Half Tablet, No Rinse	Half Tablet, with Rinse	Quarter Tablet, No Rinse	Quarter Tablet with Rinse
Marevan^®^ 2.5 mg	99.1 ± 2.4 (95.1–101.4)	99.0 ± 1.8 (96.8–101.2)	99.6 ± 3.4 (95.2–105.2)	105.9 ± 4.6 (100.3–112.0)
Warfarin Orion^®^ 2.5 mg	98.0 ± 1.1 (96.3–99.4)	96.4 ± 0.6 (95.3–97.1)	100.0 ± 2.2 (97.5–102.2)	102.1 ± 2.6 (98.8–106.2)

^1^ Samples were adjusted for the value of the control sample, i.e., a full tablet not manipulated, determined the same day. ^2^ Expected with regard to the individual fragment weight. The 5 mL oral syringe contained 2 mL purified water.
